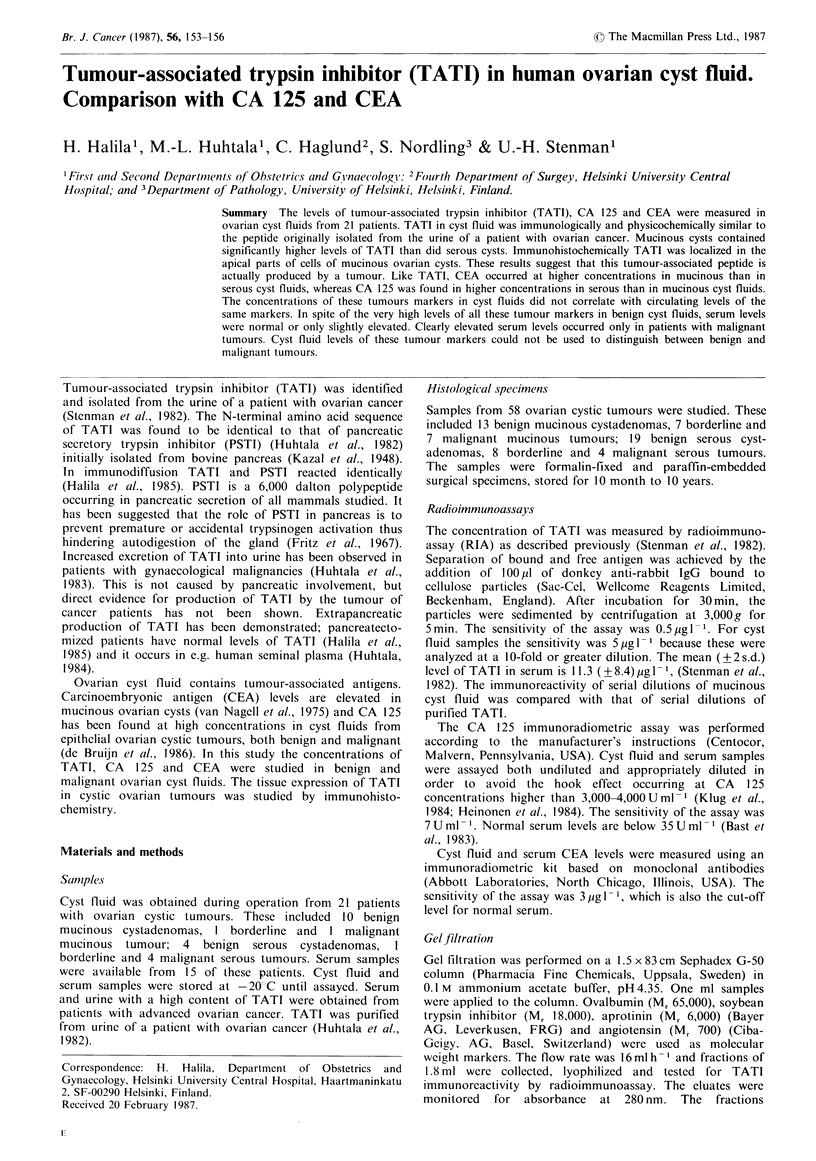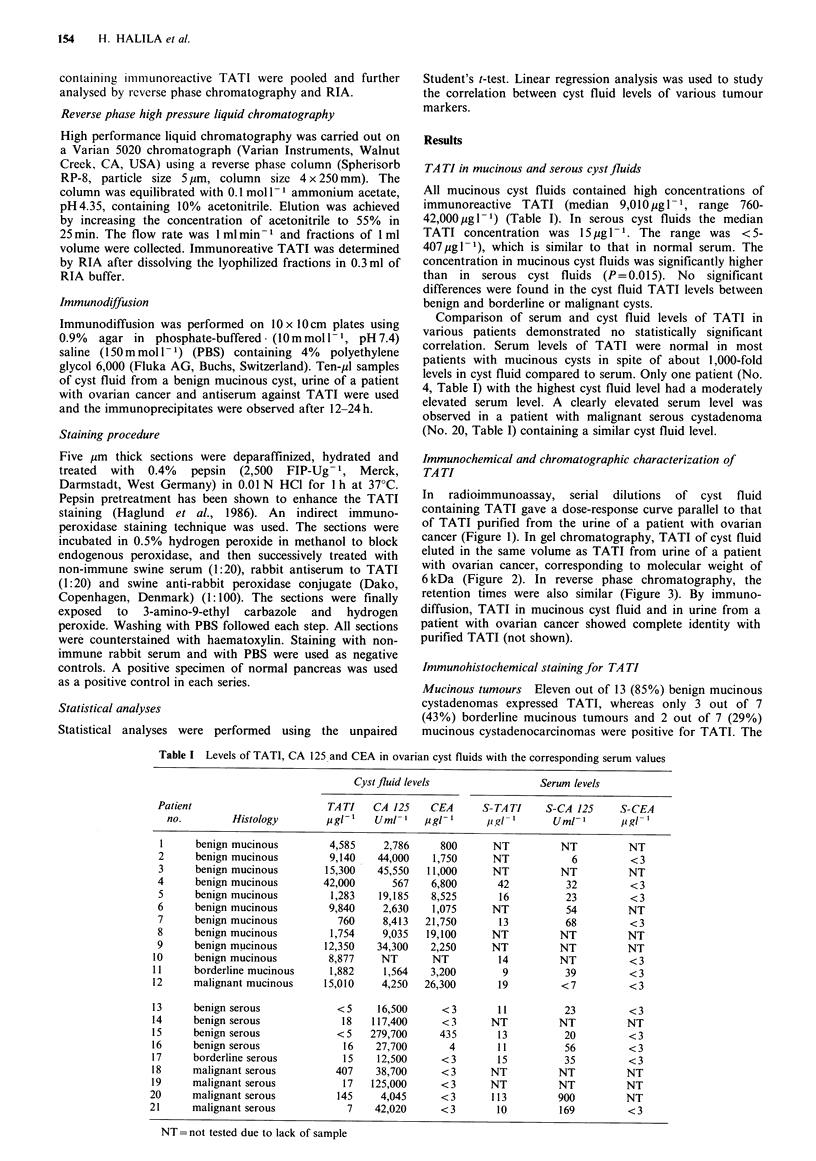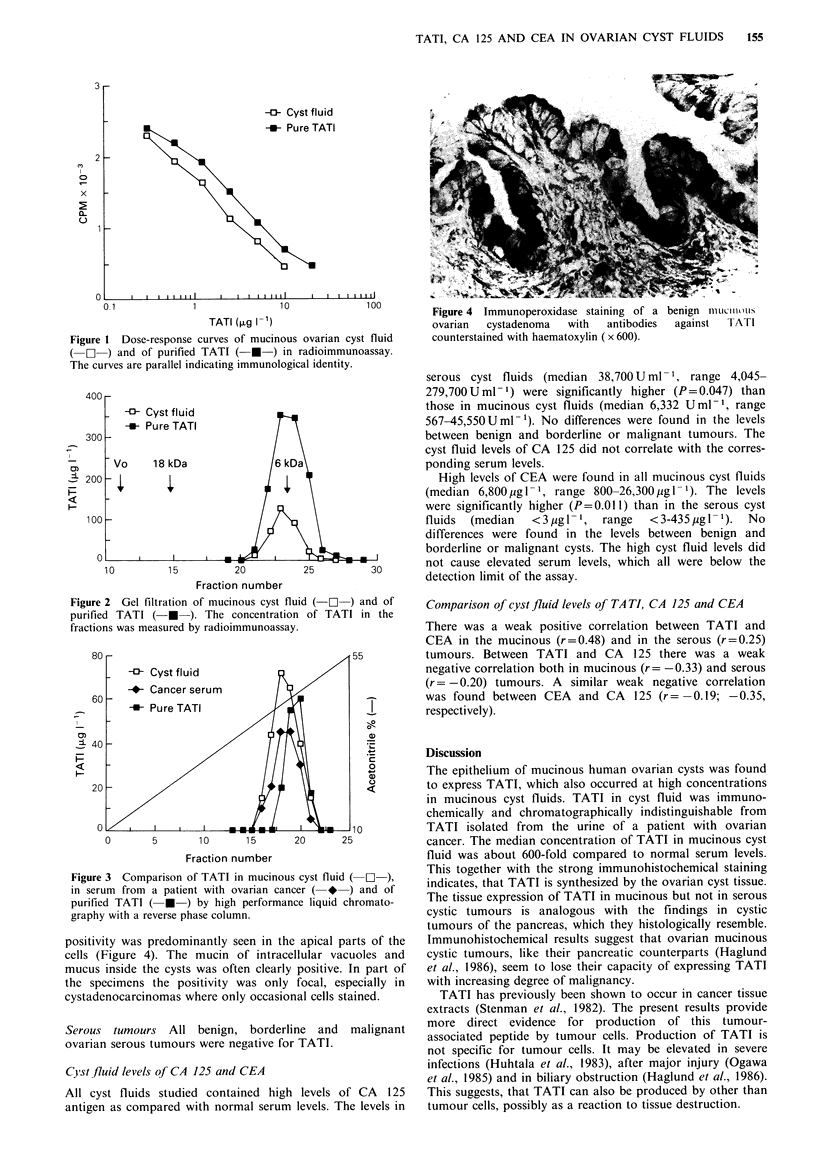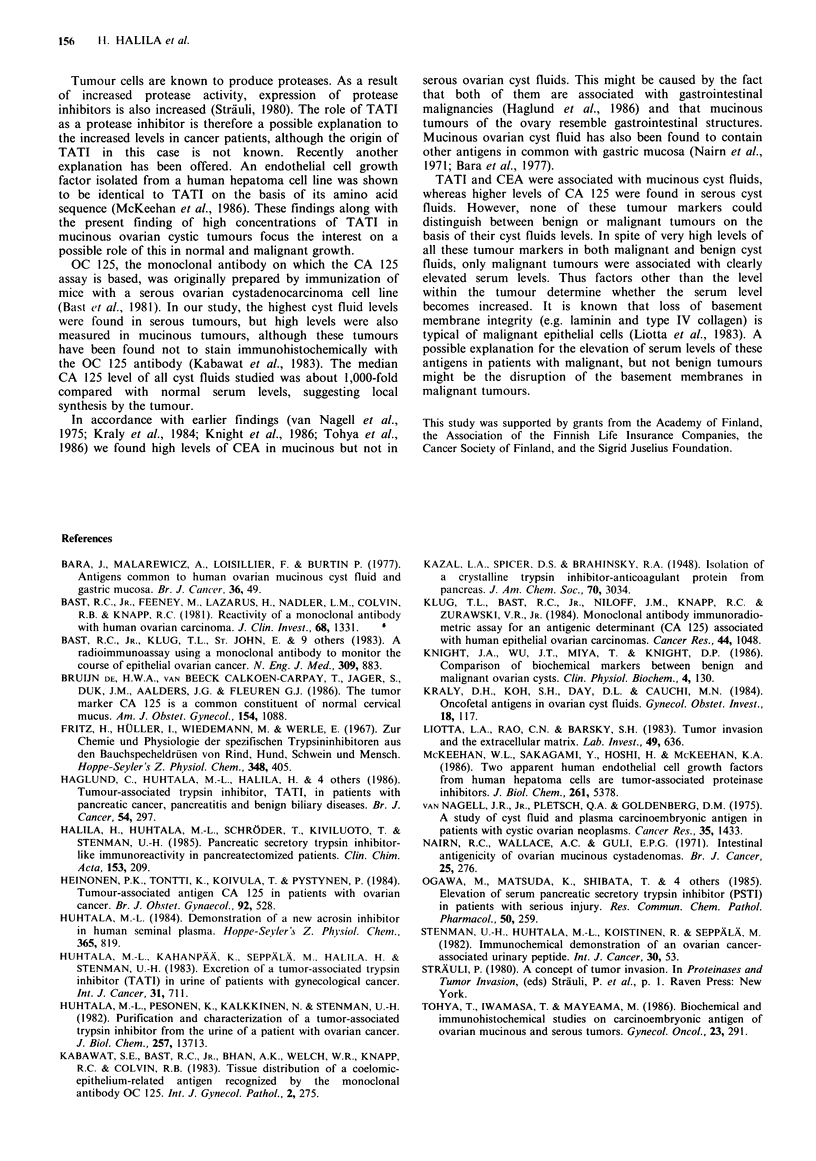# Tumour-associated trypsin inhibitor (TATI) in human ovarian cyst fluid. Comparison with CA 125 and CEA.

**DOI:** 10.1038/bjc.1987.175

**Published:** 1987-08

**Authors:** H. Halila, M. L. Huhtala, C. Haglund, S. Nordling, U. H. Stenman

**Affiliations:** First Department of Obstetrics and Gynaecology, Helsinki University Central Hospital, Finland.

## Abstract

**Images:**


Br. J. Cancer (1987), 56, 153–156 © The Macmillan Press Ltd., 1987

### Tumour-associated trypsin inhibitor (TATI) in human ovarian cyst fluid. Comparison with CA 125 and CEA

H. Halila[1], M.-L. Huhtala[1], C. Haglund[2], S. Nordling[3] & U.-H. Stenman[1]

[1]*First and Second Departments of Obstetrics and Gynaecology;* [2]*Fourth Department of Surgey, Helsinki University Central Hospital; and* [3]*Department of Pathology, University of Helsinki, Helsinki, Finland.*

**Summary** The levels of tumour-associated trypsin inhibitor (TATI), CA 125 and CEA were measured in ovarian cyst fluids from 21 patients. TATI in cyst fluid was immunologically and physicochemically similar to the peptide originally isolated from the urine of a patient with ovarian cancer. Mucinous cysts contained significantly higher levels of TATI than did serous cysts. Immunohistochemically TATI was localized in the apical parts of cells of mucinous ovarian cysts. These results suggest that this tumour-associated peptide is actually produced by a tumour. Like TATI, CEA occurred at higher concentrations in mucinous than in serous cyst fluids, whereas CA 125 was found in higher concentrations in serous than in mucinous cyst fluids. The concentrations of these tumours markers in cyst fluids did not correlate with circulating levels of the same markers. In spite of the very high levels of all these tumour markers in benign cyst fluids, serum levels were normal or only slightly elevated. Clearly elevated serum levels occurred only in patients with malignant tumours. Cyst fluid levels of these tumour markers could not be used to distinguish between benign and malignant tumours.

Tumour-associated trypsin inhibitor (TATI) was identified and isolated from the urine of a patient with ovarian cancer (Stenman *et al.*, 1982). The N-terminal amino acid sequence of TATI was found to be identical to that of pancreatic secretory trypsin inhibitor (PSTI) (Huhtala *et al.*, 1982) initially isolated from bovine pancreas (Kazal *et al.*, 1948). In immunodiffusion TATI and PSTI reacted identically (Halila *et al.*, 1985). PSTI is a 6,000 dalton polypeptide occurring in pancreatic secretion of all mammals studied. It has been suggested that the role of PSTI in pancreas is to prevent premature or accidental trypsinogen activation thus hindering autodigestion of the gland (Fritz *et al.*, 1967). Increased excretion of TATI into urine has been observed in patients with gynaecological malignancies (Huhtala *et al.*, 1983). This is not caused by pancreatic involvement, but direct evidence for production of TATI by the tumour of cancer patients has not been shown. Extrapancreatic production of TATI has been demonstrated; pancreatectomized patients have normal levels of TATI (Halila *et al.*, 1985) and it occurs in e.g. human seminal plasma (Huhtala, 1984).

Ovarian cyst fluid contains tumour-associated antigens. Carcinoembryonic antigen (CEA) levels are elevated in mucinous ovarian cysts (van Nagell *et al.*, 1975) and CA 125 has been found at high concentrations in cyst fluids from epithelial ovarian cystic tumours, both benign and malignant (de Bruijn *et al.*, 1986). In this study the concentrations of TATI, CA 125 and CEA were studied in benign and malignant ovarian cyst fluids. The tissue expression of TATI in cystic ovarian tumours was studied by immunohistochemistry.

### Materials and methods

### *Samples*

Cyst fluid was obtained during operation from 21 patients with ovarian cystic tumours. These included 10 benign mucinous cystadenomas, 1 borderline and 1 malignant mucinous tumour; 4 benign serous cystadenomas, 1 borderline and 4 malignant serous tumours. Serum samples were available from 15 of these patients. Cyst fluid and serum samples were stored at $-20^{\circ}$C until assayed. Serum and urine with a high content of TATI were obtained from patients with advanced ovarian cancer. TATI was purified from urine of a patient with ovarian cancer (Huhtala *et al.*, 1982).

Correspondence: H. Halila, Department of Obstetrics and Gynaecology, Helsinki University Central Hospital, Haartmaninkatu 2, SF-00290 Helsinki, Finland.
Received 20 February 1987.

### *Histological specimens*

Samples from 58 ovarian cystic tumours were studied. These included 13 benign mucinous cystadenomas, 7 borderline and 7 malignant mucinous tumours; 19 benign serous cystadenomas, 8 borderline and 4 malignant serous tumours. The samples were formalin-fixed and paraffin-embedded surgical specimens, stored for 10 month to 10 years.

### *Radioimmunoassays*

The concentration of TATI was measured by radioimmunoassay (RIA) as described previously (Stenman *et al.*, 1982). Separation of bound and free antigen was achieved by the addition of 100 $\mu$l of donkey anti-rabbit IgG bound to cellulose particles (Sac-Cel, Wellcome Reagents Limited, Beckenham, England). After incubation for 30 min, the particles were sedimented by centrifugation at 3,000 *g* for 5 min. The sensitivity of the assay was 0.5 $\mu g\,l^{-1}$. For cyst fluid samples the sensitivity was 5 $\mu g\,l^{-1}$ because these were analyzed at a 10-fold or greater dilution. The mean ($\pm 2$ s.d.) level of TATI in serum is 11.3 ($\pm 8.4$) $\mu g\,l^{-1}$, (Stenman *et al.*, 1982). The immunoreactivity of serial dilutions of mucinous cyst fluid was compared with that of serial dilutions of purified TATI.

The CA 125 immunoradiometric assay was performed according to the manufacturer's instructions (Centocor, Malvern, Pennsylvania, USA). Cyst fluid and serum samples were assayed both undiluted and appropriately diluted in order to avoid the hook effect occurring at CA 125 concentrations higher than 3,000–4,000 U ml$^{-1}$ (Klug *et al.*, 1984; Heinonen *et al.*, 1984). The sensitivity of the assay was 7 U ml$^{-1}$. Normal serum levels are below 35 U ml$^{-1}$ (Bast *et al.*, 1983).

Cyst fluid and serum CEA levels were measured using an immunoradiometric kit based on monoclonal antibodies (Abbott Laboratories, North Chicago, Illinois, USA). The sensitivity of the assay was 3 $\mu g\,l^{-1}$, which is also the cut-off level for normal serum.

### *Gel filtration*

Gel filtration was performed on a 1.5 × 83 cm Sephadex G-50 column (Pharmacia Fine Chemicals, Uppsala, Sweden) in 0.1 M ammonium acetate buffer, pH 4.35. One ml samples were applied to the column. Ovalbumin ($M_r$ 65,000), soybean trypsin inhibitor ($M_r$ 18,000), aprotinin ($M_r$ 6,000) (Bayer AG, Leverkusen, FRG) and angiotensin ($M_r$ 700) (Ciba-Geigy, AG, Basel, Switzerland) were used as molecular weight markers. The flow rate was 16 ml h$^{-1}$ and fractions of 1.8 ml were collected, lyophilized and tested for TATI immunoreactivity by radioimmunoassay. The eluates were monitored for absorbance at 280 nm. The fractions containing immunoreactive TATI were pooled and further analysed by reverse phase chromatography and RIA.

*Reverse phase high pressure liquid chromatography*

High performance liquid chromatography was carried out on a Varian 5020 chromatograph (Varian Instruments, Walnut Creek, CA, USA) using a reverse phase column (Spherisorb RP-8, particle size 5 $\mu$m, column size 4 × 250 mm). The column was equilibrated with 0.1 mol l$^{-1}$ ammonium acetate, pH 4.35, containing 10% acetonitrile. Elution was achieved by increasing the concentration of acetonitrile to 55% in 25 min. The flow rate was 1 ml min$^{-1}$ and fractions of 1 ml volume were collected. Immunoreative TATI was determined by RIA after dissolving the lyophilized fractions in 0.3 ml of RIA buffer.

*Immunodiffusion*

Immunodiffusion was performed on 10 × 10 cm plates using 0.9% agar in phosphate-buffered (10 m mol l$^{-1}$, pH 7.4) saline (150 m mol l$^{-1}$) (PBS) containing 4% polyethylene glycol 6,000 (Fluka AG, Buchs, Switzerland). Ten-$\mu$l samples of cyst fluid from a benign mucinous cyst, urine of a patient with ovarian cancer and antiserum against TATI were used and the immunoprecipitates were observed after 12–24 h.

*Staining procedure*

Five $\mu$m thick sections were deparaffinized, hydrated and treated with 0.4% pepsin (2,500 FIP-Ug$^{-1}$, Merck, Darmstadt, West Germany) in 0.01 N HCl for 1 h at 37°C. Pepsin pretreatment has been shown to enhance the TATI staining (Haglund *et al.*, 1986). An indirect immunoperoxidase staining technique was used. The sections were incubated in 0.5% hydrogen peroxide in methanol to block endogenous peroxidase, and then successively treated with non-immune swine serum (1:20), rabbit antiserum to TATI (1:20) and swine anti-rabbit peroxidase conjugate (Dako, Copenhagen, Denmark) (1:100). The sections were finally exposed to 3-amino-9-ethyl carbazole and hydrogen peroxide. Washing with PBS followed each step. All sections were counterstained with haematoxylin. Staining with non-immune rabbit serum and with PBS were used as negative controls. A positive specimen of normal pancreas was used as a positive control in each series.

*Statistical analyses*

Statistical analyses were performed using the unpaired Student's *t*-test. Linear regression analysis was used to study the correlation between cyst fluid levels of various tumour markers.

### Results

*TATI in mucinous and serous cyst fluids*

All mucinous cyst fluids contained high concentrations of immunoreactive TATI (median 9,010 $\mu$g l$^{-1}$, range 760-42,000 $\mu$g l$^{-1}$) (Table I). In serous cyst fluids the median TATI concentration was 15 $\mu$g l$^{-1}$. The range was <5-407 $\mu$g l$^{-1}$), which is similar to that in normal serum. The concentration in mucinous cyst fluids was significantly higher than in serous cyst fluids ($P = 0.015$). No significant differences were found in the cyst fluid TATI levels between benign and borderline or malignant cysts.

Comparison of serum and cyst fluid levels of TATI in various patients demonstrated no statistically significant correlation. Serum levels of TATI were normal in most patients with mucinous cysts in spite of about 1,000-fold levels in cyst fluid compared to serum. Only one patient (No. 4, Table I) with the highest cyst fluid level had a moderately elevated serum level. A clearly elevated serum level was observed in a patient with malignant serous cystadenoma (No. 20, Table I) containing a similar cyst fluid level.

*Immunochemical and chromatographic characterization of TATI*

In radioimmunoassay, serial dilutions of cyst fluid containing TATI gave a dose-response curve parallel to that of TATI purified from the urine of a patient with ovarian cancer (Figure 1). In gel chromatography, TATI of cyst fluid eluted in the same volume as TATI from urine of a patient with ovarian cancer, corresponding to molecular weight of 6 kDa (Figure 2). In reverse phase chromatography, the retention times were also similar (Figure 3). By immunodiffusion, TATI in mucinous cyst fluid and in urine from a patient with ovarian cancer showed complete identity with purified TATI (not shown).

*Immunohistochemical staining for TATI*

*Mucinous tumours* Eleven out of 13 (85%) benign mucinous cystadenomas expressed TATI, whereas only 3 out of 7 (43%) borderline mucinous tumours and 2 out of 7 (29%) mucinous cystadenocarcinomas were positive for TATI. The

**Table I** Levels of TATI, CA 125 and CEA in ovarian cyst fluids with the corresponding serum values

| | | *Cyst fluid levels* | | | *Serum levels* | | |
|---|---|---|---|---|---|---|---|
| *Patient no.* | *Histology* | *TATI* $\mu g l^{-1}$ | *CA 125* $U ml^{-1}$ | *CEA* $\mu g l^{-1}$ | *S-TATI* $\mu g l^{-1}$ | *S-CA 125* $U ml^{-1}$ | *S-CEA* $\mu g l^{-1}$ |
| 1 | benign mucinous | 4,585 | 2,786 | 800 | NT | NT | NT |
| 2 | benign mucinous | 9,140 | 44,000 | 1,750 | NT | 6 | <3 |
| 3 | benign mucinous | 15,300 | 45,550 | 11,000 | NT | NT | NT |
| 4 | benign mucinous | 42,000 | 567 | 6,800 | 42 | 32 | <3 |
| 5 | benign mucinous | 1,283 | 19,185 | 8,525 | 16 | 23 | <3 |
| 6 | benign mucinous | 9,840 | 2,630 | 1,075 | NT | 54 | NT |
| 7 | benign mucinous | 760 | 8,413 | 21,750 | 13 | 68 | <3 |
| 8 | benign mucinous | 1,754 | 9,035 | 19,100 | NT | NT | NT |
| 9 | benign mucinous | 12,350 | 34,300 | 2,250 | NT | NT | NT |
| 10 | benign mucinous | 8,877 | NT | NT | 14 | NT | <3 |
| 11 | borderline mucinous | 1,882 | 1,564 | 3,200 | 9 | 39 | <3 |
| 12 | malignant mucinous | 15,010 | 4,250 | 26,300 | 19 | <7 | <3 |
| 13 | benign serous | <5 | 16,500 | <3 | 11 | 23 | <3 |
| 14 | benign serous | 18 | 117,400 | <3 | NT | NT | NT |
| 15 | benign serous | <5 | 279,700 | 435 | 13 | 20 | <3 |
| 16 | benign serous | 16 | 27,700 | 4 | 11 | 56 | <3 |
| 17 | borderline serous | 15 | 12,500 | <3 | 15 | 35 | <3 |
| 18 | malignant serous | 407 | 38,700 | <3 | NT | NT | NT |
| 19 | malignant serous | 17 | 125,000 | <3 | NT | NT | NT |
| 20 | malignant serous | 145 | 4,045 | <3 | 113 | 900 | NT |
| 21 | malignant serous | 7 | 42,020 | <3 | 10 | 169 | <3 |

NT = not tested due to lack of sample

**Figure 1** Dose-response curves of mucinous ovarian cyst fluid (—□—) and of purified TATI (—■—) in radioimmunoassay. The curves are parallel indicating immunological identity.

**Figure 2** Gel filtration of mucinous cyst fluid (—□—) and of purified TATI (—■—). The concentration of TATI in the fractions was measured by radioimmunoassay.

**Figure 3** Comparison of TATI in mucinous cyst fluid (—□—), in serum from a patient with ovarian cancer (—◆—) and of purified TATI (—■—) by high performance liquid chromatography with a reverse phase column.

positivity was predominantly seen in the apical parts of the cells (Figure 4). The mucin of intracellular vacuoles and mucus inside the cysts was often clearly positive. In part of the specimens the positivity was only focal, especially in cystadenocarcinomas where only occasional cells stained.

*Serous tumours* All benign, borderline and malignant ovarian serous tumours were negative for TATI.

*Cyst fluid levels of CA 125 and CEA*

All cyst fluids studied contained high levels of CA 125 antigen as compared with normal serum levels. The levels in serous cyst fluids (median 38,700 U ml$^{-1}$, range 4,045–279,700 U ml$^{-1}$) were significantly higher ($P=0.047$) than those in mucinous cyst fluids (median 6,332 U ml$^{-1}$, range 567–45,550 U ml$^{-1}$). No differences were found in the levels between benign and borderline or malignant tumours. The cyst fluid levels of CA 125 did not correlate with the corresponding serum levels.

**Figure 4** Immunoperoxidase staining of a benign mucinous ovarian cystadenoma with antibodies against TATI counterstained with haematoxylin (×600).

High levels of CEA were found in all mucinous cyst fluids (median 6,800 μg l$^{-1}$, range 800–26,300 μg l$^{-1}$). The levels were significantly higher ($P=0.011$) than in the serous cyst fluids (median <3 μg l$^{-1}$, range <3-435 μg l$^{-1}$). No differences were found in the levels between benign and borderline or malignant cysts. The high cyst fluid levels did not cause elevated serum levels, which all were below the detection limit of the assay.

*Comparison of cyst fluid levels of TATI, CA 125 and CEA*

There was a weak positive correlation between TATI and CEA in the mucinous ($r=0.48$) and in the serous ($r=0.25$) tumours. Between TATI and CA 125 there was a weak negative correlation both in mucinous ($r=-0.33$) and serous ($r=-0.20$) tumours. A similar weak negative correlation was found between CEA and CA 125 ($r=-0.19$; $-0.35$, respectively).

### Discussion

The epithelium of mucinous human ovarian cysts was found to express TATI, which also occurred at high concentrations in mucinous cyst fluids. TATI in cyst fluid was immunochemically and chromatographically indistinguishable from TATI isolated from the urine of a patient with ovarian cancer. The median concentration of TATI in mucinous cyst fluid was about 600-fold compared to normal serum levels. This together with the strong immunohistochemical staining indicates, that TATI is synthesized by the ovarian cyst tissue. The tissue expression of TATI in mucinous but not in serous cystic tumours is analogous with the findings in cystic tumours of the pancreas, which they histologically resemble. Immunohistochemical results suggest that ovarian mucinous cystic tumours, like their pancreatic counterparts (Haglund *et al.*, 1986), seem to lose their capacity of expressing TATI with increasing degree of malignancy.

TATI has previously been shown to occur in cancer tissue extracts (Stenman *et al.*, 1982). The present results provide more direct evidence for production of this tumour-associated peptide by tumour cells. Production of TATI is not specific for tumour cells. It may be elevated in severe infections (Huhtala *et al.*, 1983), after major injury (Ogawa *et al.*, 1985) and in biliary obstruction (Haglund *et al.*, 1986). This suggests, that TATI can also be produced by other than tumour cells, possibly as a reaction to tissue destruction.

Tumour cells are known to produce proteases. As a result of increased protease activity, expression of protease inhibitors is also increased (Sträuli, 1980). The role of TATI as a protease inhibitor is therefore a possible explanation to the increased levels in cancer patients, although the origin of TATI in this case is not known. Recently another explanation has been offered. An endothelial cell growth factor isolated from a human hepatoma cell line was shown to be identical to TATI on the basis of its amino acid sequence (McKeehan *et al.*, 1986). These findings along with the present finding of high concentrations of TATI in mucinous ovarian cystic tumours focus the interest on a possible role of this in normal and malignant growth.

OC 125, the monoclonal antibody on which the CA 125 assay is based, was originally prepared by immunization of mice with a serous ovarian cystadenocarcinoma cell line (Bast *et al.*, 1981). In our study, the highest cyst fluid levels were found in serous tumours, but high levels were also measured in mucinous tumours, although these tumours have been found not to stain immunohistochemically with the OC 125 antibody (Kabawat *et al.*, 1983). The median CA 125 level of all cyst fluids studied was about 1,000-fold compared with normal serum levels, suggesting local synthesis by the tumour.

In accordance with earlier findings (van Nagell *et al.*, 1975; Kraly *et al.*, 1984; Knight *et al.*, 1986; Tohya *et al.*, 1986) we found high levels of CEA in mucinous but not in serous ovarian cyst fluids. This might be caused by the fact that both of them are associated with gastrointestinal malignancies (Haglund *et al.*, 1986) and that mucinous tumours of the ovary resemble gastrointestinal structures. Mucinous ovarian cyst fluid has also been found to contain other antigens in common with gastric mucosa (Nairn *et al.*, 1971; Bara *et al.*, 1977).

TATI and CEA were associated with mucinous cyst fluids, whereas higher levels of CA 125 were found in serous cyst fluids. However, none of these tumour markers could distinguish between benign or malignant tumours on the basis of their cyst fluids levels. In spite of very high levels of all these tumour markers in both malignant and benign cyst fluids, only malignant tumours were associated with clearly elevated serum levels. Thus factors other than the level within the tumour determine whether the serum level becomes increased. It is known that loss of basement membrane integrity (e.g. laminin and type IV collagen) is typical of malignant epithelial cells (Liotta *et al.*, 1983). A possible explanation for the elevation of serum levels of these antigens in patients with malignant, but not benign tumours might be the disruption of the basement membranes in malignant tumours.

This study was supported by grants from the Academy of Finland, the Association of the Finnish Life Insurance Companies, the Cancer Society of Finland, and the Sigrid Juselius Foundation.

### References

BARA, J., MALAREWICZ, A., LOISILLIER, F. & BURTIN P. (1977). Antigens common to human ovarian mucinous cyst fluid and gastric mucosa. *Br. J. Cancer*, **36**, 49.

BAST, R.C., JR., FEENEY, M., LAZARUS, H., NADLER, L.M., COLVIN, R.B. & KNAPP, R.C. (1981). Reactivity of a monoclonal antibody with human ovarian carcinoma. *J. Clin. Invest.*, **68**, 1331.

BAST, R.C., JR., KLUG, T.L., ST. JOHN, E. & 9 others (1983). A radioimmunoassay using a monoclonal antibody to monitor the course of epithelial ovarian cancer. *N. Eng. J. Med.*, **309**, 883.

BRUIJN DE, H.W.A., VAN BEECK CALKOEN-CARPAY, T., JAGER, S., DUK, J.M., AALDERS, J.G. & FLEUREN G.J. (1986). The tumor marker CA 125 is a common constituent of normal cervical mucus. *Am. J. Obstet. Gynecol.*, **154**, 1088.

FRITZ, H., HÜLLER, I., WIEDEMANN, M. & WERLE, E. (1967). Zur Chemie und Physiologie der spezifischen Trypsininhibitoren aus den Bauchspecheldrüsen von Rind, Hund, Schwein und Mensch. *Hoppe-Seyler's Z. Physiol. Chem.*, **348**, 405.

HAGLUND, C., HUHTALA, M.-L., HALILA, H. & 4 others (1986). Tumour-associated trypsin inhibitor, TATI, in patients with pancreatic cancer, pancreatitis and benign biliary diseases. *Br. J. Cancer*, **54**, 297.

HALILA, H., HUHTALA, M.-L., SCHRÖDER, T., KIVILUOTO, T. & STENMAN, U.-H. (1985). Pancreatic secretory trypsin inhibitor-like immunoreactivity in pancreatectomized patients. *Clin. Chim. Acta*, **153**, 209.

HEINONEN, P.K., TONTTI, K., KOIVULA, T. & PYSTYNEN, P. (1984). Tumour-associated antigen CA 125 in patients with ovarian cancer. *Br. J. Obstet. Gynaecol.*, **92**, 528.

HUHTALA, M.-L. (1984). Demonstration of a new acrosin inhibitor in human seminal plasma. *Hoppe-Seyler's Z. Physiol. Chem.*, **365**, 819.

HUHTALA, M.-L., KAHANPÄÄ, K., SEPPÄLÄ, M., HALILA, H. & STENMAN, U.-H. (1983). Excretion of a tumor-associated trypsin inhibitor (TATI) in urine of patients with gynecological cancer. *Int. J. Cancer*, **31**, 711.

HUHTALA, M.-L., PESONEN, K., KALKKINEN, N. & STENMAN, U.-H. (1982). Purification and characterization of a tumor-associated trypsin inhibitor from the urine of a patient with ovarian cancer. *J. Biol. Chem.*, **257**, 13713.

KABAWAT, S.E., BAST, R.C., JR., BHAN, A.K., WELCH, W.R., KNAPP, R.C. & COLVIN, R.B. (1983). Tissue distribution of a coelomic-epithelium-related antigen recognized by the monoclonal antibody OC 125. *Int. J. Gynecol. Pathol.*, **2**, 275.

KAZAL, L.A., SPICER, D.S. & BRAHINSKY, R.A. (1948). Isolation of a crystalline trypsin inhibitor-anticoagulant protein from pancreas. *J. Am. Chem. Soc.*, **70**, 3034.

KLUG, T.L., BAST, R.C., JR., NILOFF, J.M., KNAPP, R.C. & ZURAWSKI, V.R., JR. (1984). Monoclonal antibody immunoradiometric assay for an antigenic determinant (CA 125) associated with human epithelial ovarian carcinomas. *Cancer Res.*, **44**, 1048.

KNIGHT, J.A., WU, J.T., MIYA, T. & KNIGHT, D.P. (1986). Comparison of biochemical markers between benign and malignant ovarian cysts. *Clin. Physiol. Biochem.*, **4**, 130.

KRALY, D.H., KOH, S.H., DAY, D.L. & CAUCHI, M.N. (1984). Oncofetal antigens in ovarian cyst fluids. *Gynecol. Obstet. Invest.*, **18**, 117.

LIOTTA, L.A., RAO, C.N. & BARSKY, S.H. (1983). Tumor invasion and the extracellular matrix. *Lab. Invest.*, **49**, 636.

McKEEHAN, W.L., SAKAGAMI, Y., HOSHI, H. & McKEEHAN, K.A. (1986). Two apparent human endothelial cell growth factors from human hepatoma cells are tumor-associated proteinase inhibitors. *J. Biol. Chem.*, **261**, 5378.

VAN NAGELL, J.R., JR., PLETSCH, Q.A. & GOLDENBERG, D.M. (1975). A study of cyst fluid and plasma carcinoembryonic antigen in patients with cystic ovarian neoplasms. *Cancer Res.*, **35**, 1433.

NAIRN, R.C., WALLACE, A.C. & GULI, E.P.G. (1971). Intestinal antigenicity of ovarian mucinous cystadenomas. *Br. J. Cancer*, **25**, 276.

OGAWA, M., MATSUDA, K., SHIBATA, T. & 4 others (1985). Elevation of serum pancreatic secretory trypsin inhibitor (PSTI) in patients with serious injury. *Res. Commun. Chem. Pathol. Pharmacol.*, **50**, 259.

STENMAN, U.-H., HUHTALA, M.-L., KOISTINEN, R. & SEPPÄLÄ, M. (1982). Immunochemical demonstration of an ovarian cancer-associated urinary peptide. *Int. J. Cancer*, **30**, 53.

STRÄULI, P. (1980). A concept of tumor invasion. In *Proteinases and Tumor Invasion*, (eds) Sträuli, P. *et al.*, p. 1. Raven Press: New York.

TOHYA, T., IWAMASA, T. & MAYEAMA, M. (1986). Biochemical and immunohistochemical studies on carcinoembryonic antigen of ovarian mucinous and serous tumors. *Gynecol. Oncol.*, **23**, 291.